# Does vancomycin administered at an empirical dose ensure coverage of pediatric patients against gram-positive pathogens?

**DOI:** 10.5935/0103-507X.20200067

**Published:** 2020

**Authors:** Frederico Ribeiro Pires, Stefano Ivani de Paula, Artur Figueiredo Delgado, Werther Brunow de Carvalho, Nilo José Coelho Duarte, Ronaldo Morales Júnior, Silvia Regina Cavani Jorge Santos

**Affiliations:** 1 Center for Pediatric Intensive Care, Hospital das Clínicas, Faculdade de Medicina, Universidade de São Paulo - São Paulo (SP), Brazil.; 2 Central Laboratory Division, Central Institute, Hospital das Clínicas, Faculdade de Medicina, Universidade de São Paulo - São Paulo (SP), Brazil.; 3 Center of Clinical Pharmacokinetics, Faculdade de Ciências Farmacêuticas, Universidade de São Paulo - São Paulo (SP), Brazil.

**Keywords:** Vancomycin/administration & dosage, Drug monitoring, Pharmacokinetics, Pharmacologic actions, Pediatric intensive care units, Child, Vancomicina, Monitoramento de medicamentos, Farmacocinética, Ações farmacológicas, Unidades de terapia intensiva pediátrica, Criança

## Abstract

**Objective:**

To investigate the vancomycin effectiveness against gram-positive pathogens with the minimum inhibitory concentration of 1mg/L in pediatric patients based on the area under the curve and the minimum inhibitory concentration ratio > 400.

**Methods:**

A population of 22 pediatric patients (13 boys) admitted to the pediatric intensive care unit with preserved renal function was stratified in two groups (G1 < 7 years and G2 ≥ 7 years). After the fourth dose administered of vancomycin (10 - 15mg/kg every 6 hours) was administered, two blood samples were collected (third and fifth hours), followed by serum measurement by immunoassays to investigate the pharmacokinetics and antimicrobial coverage.

**Results:**

There was no difference between the groups regarding dose, trough level or area under the curve. Coverage against gram-positive pathogens with a minimum inhibitory concentration of 1mg/L occurred in only 46% of patients in both groups. The pharmacokinetics in both groups were altered relative to the reference values, and the groups differed in regard to increased total body clearance and shortening of the biological half-life, which were more pronounced in younger patients.

**Conclusion:**

A minimum empirical dose of 60mg/kg per day should be prescribed for pediatric patients in intensive care units with preserved renal function. The use of the ratio between the area under the curve and minimum inhibitory concentration in the evaluation of vancomycin coverage is recommended to achieve the desired outcome, since the pharmacokinetics are altered in these patients, which may impact the effectiveness of the antimicrobial.

## Abstract (pt)

**Objetivo:**

Investigar a efetividade da vancomicina contra Gram-positivos com concentração inibitória mínima de 1mg/L em pacientes pediátricos com base na razão entre área sob a curva e concentração inibitória mínima > 400.

**Métodos:**

População de 22 pacientes pediátricos (13 meninos) internados no centro de terapia intensiva pediátrica, com função renal preservada, que foram distribuídos em dois grupos (G1 < 7 anos e G2 ≥ 7 anos). Após a quarta dose de vancomicina (10 - 15mg/kg a cada 6 horas), duas amostras de sangue foram colhidas (terceira e quinta horas), seguidas da dosagem sérica por imunoensaios para investigação da farmacocinética e da cobertura do antimicrobiano.

**Resultados:**

Não se registrou diferença entre os grupos com relação à dose, ao nível de vale ou ainda na área sob a curva. A cobertura contra Gram-positivos com concentração inibitória mínima de 1mg/L ocorreu em apenas 46% dos pacientes em ambos os grupos. A farmacocinética se mostrou alterada nos dois grupos diante dos valores de referência, mas a diferença entre grupos foi registrada pelo aumento da depuração total corporal e pelo encurtamento da meia-vida biológica, mais pronunciados nos pacientes mais novos.

**Conclusão:**

A dose empírica mínima de 60mg/kg ao dia deve ser prescrita ao paciente pediátrico de unidade de terapia intensiva com função renal preservada. A utilização da razão entre área sob a curva e concentração inibitória mínima na avaliação da cobertura da vancomicina é recomendada para se atingir o desfecho desejado, uma vez que a farmacocinética está alterada nesses pacientes, podendo impactar na efetividade do antimicrobiano.

## INTRODUCTION

Vancomycin is a complex tricyclic glycopeptide that inhibits cell wall synthesis.^(1)^ According to the main consensuses, it is widely prescribed to patients in intensive care for the treatment of severe infections such as meningitis, osteomyelitis, endocarditis, bloodstream infection, pneumonia and skin and soft tissue infections.^(2,3)^ There has been a growing increase in the prevalence of infections caused by gram-positive bacteria, especially methicillin*-*resistant *Staphylococcus aureus* (MRSA), in the hospital environment due to the widespread use of this antimicrobial.^(4)^

Until 2011, the Infectious Diseases Society of America (IDSA) recommended therapeutic monitoring via measurement of the trough serum vancomycin concentration in critically ill adult and pediatric patients with infection caused by nosocomial pathogens.^(3)^ This is because an adequate serum level of vancomycin is necessary to achieve a bactericidal serum level and is associated with a lower rate of treatment failure and a lower risk of developing bacterial resistance; thus, the measurement of serum levels is important. The recommended pediatric dose is 10 to 15mg/kg every 6 hours, with a strong recommendation for the use of the highest value to control the most severe infections, based on data extrapolated from adult patients, to achieve a trough serum vancomycin level of 15 - 20mg/L.^(5)^

Currently, the target is related to the ratio of the area under the steady-state serum vancomycin concentration curve over the period of 0 - 24 hours (AUC^ss^_0-24_) to the minimum inhibitory concentration (MIC) of the isolated pathogen. This objective aims to correlate the therapeutic target with the recommended AUC-to-MIC ratio (AUC^ss^_0-24_/MIC) > 400, which is related to the effectiveness of vancomycin.^(3)^ An AUC^ss^_0-24_/MIC > 400 is the recommended pharmacodynamic/pharmacokinetic target for the treatment of *S. aureus* infection with intravenous vancomycin based on *in vitro* animal and human studies.^(6)^

Numerous studies conducted with pediatric patients in intensive care have shown that the trough serum level varies widely as a function of age and renal function. Among other causes of error, those related to the administration of the medication stand out. Attention must be paid to such aspects as following the administration schedule, using an infusion pump, collecting blood samples adequately and adhering to the recommended collection times to obtain viable samples for serum monitoring and to ensure the reliability of the results. Errors in these areas lead to low reliability in the assessment of vancomycin efficacy in pediatric patients in intensive care when blood is collected for the measurement of the trough serum concentration.^(7-10)^ Based on this real problem, we conducted this study to assess the effectiveness of vancomycin in pediatric patients based on AUC^ss^_0-24_/MIC > 400 by standardizing blood sample collection at the recommended strategic times and considering the peculiarities of the large age group of patients in a pediatric intensive care unit (ICU).

## METHODS

This is a pilot, observational, single-center study conducted between November 2017 and March 2018 that included 22 pediatric patients admitted to the Intensive Care Center of the Children’s Institute of *Hospital das Clínicas da Faculdade de Medicina* of the *Universidade de São Paulo* (USP). The study was approved by the Research Ethics Committee of the hospital under CAAE no. 81995318.8.0000.0068.

Pediatric patients of both sexes were included (13 boys). The patients were between 1.5 and 15.3 years of age and were admitted to the Intensive Care Center with indications for vancomycin at the discretion of the physicians on duty. All patients had preserved renal function (creatinine clearance - ClCr > 90mL/min per 1.73m^2^) according to the Schwartz equation, which is calculated taking into account serum creatinine (urease and glutamate dehydrogenase enzymatic kinetic method), patient height and a pre-established constant based on the age group. Patients who were admitted to the service with previous use of vancomycin or with altered renal function were excluded. The study population was divided into two equal groups based on age - younger or older than 7 years - for the comparison of pharmacokinetic and pharmacodynamic responses given that drug clearance differs by age; that is, clearance decreases the older the child is.^(4,9)^ A total of 11 patients aged < 7 years were allocated to Group 1 (G1), and 11 patients aged ≥ 7 years were allocated to Group 2 (G2).

Patients were included in the groups after detailed information on the study protocol was provided to the legal guardian, who granted authorization for participation by signing the informed consent form. After consent was obtained, each patient’s form was filled with epidemiological data and clinical data on the disease, renal function, reason for using the antimicrobial, actual weight, ideal weight (W_50_ in the curve for height) and other relevant information. Because this was an observational study, the vancomycin dose was prescribed at the discretion of the attending physician at the recommended daily dose of 40 to 60mg/kg (10 to 15mg/kg every 6 hours) and was administered by infusion pump for 1 hour. After the fourth dose of the antimicrobial (steady state), two blood samples were collected (1mL/each); the first collection was performed at the third hour after the beginning of the infusion, and the second was performed at the fifth hour after the beginning of the infusion for the measurement of serum levels by immunoassays with the commercial TDX kit (polarized fluorescence immunoassays). The collection times were chosen to avoid measurement errors caused by serum levels obtained before the distribution equilibrium, which occurs when collection takes place in the first and second hours. The two collections can be performed any time between the third and sixth hours; thus, blood collections were performed in the third and fifth hours.

### Statistical analysis

The single-compartment model was applied to study the pharmacokinetics of vancomycin based on the simplification performed with the noncompartmental data analysis software package PK Solutions 2.0 (Summit, USA). The parameters related to elimination were calculated; these include the elimination rate (kel), biological half-life (t_(1/2) β_) and plasma clearance or total body clearance (CL_T_), the latter of which is characterized as the speed of drug clearance by an organism. The extent of distribution was expressed as the apparent volume of distribution (Vd^ss^). As the rate at which the serum level decreased between measurements was individually assessed, these parameters were calculated for each patient, thus conferring uniqueness to the data. This allowed a consideration of the interaction of the results with the actual situation of each patient.

The programs Excel 2007 (Microsoft Corporation, USA) and GraphPad Prism v.5.0 (GraphPad Software, Inc., San Diego, CA, USA) were used to organize and present population data based on the individual data obtained and to calculate the statistical parameters for comparisons between groups and for the correlation analysis. Parametric and nonparametric statistics were applied in the correlation analysis, and p < 0.05 was considered statistically significant. The data related to the demographic characteristics of the patient population, as well as the results related to the dose, serum levels, pharmacokinetic analysis and the pharmacokinetic/pharmacodynamic (PK/PD) approach, are expressed as medians (interquartile range - IQR) because nonparametric statistics were applied and because a small number of outliers were observed, which could distort the mean.

## RESULTS

**Figure 1:**
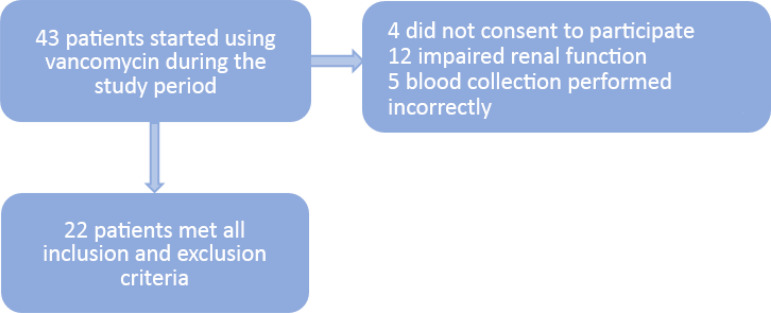
Characterization of the population after inclusion and exclusion criteria.

Considering the inclusion and exclusion criteria of the study, 22 patients were selected (Figure 1 and Table 1S - Supplementary material); they had a median age of 9.5 (IQR 3.5 - 11.8), a minimum age of 1.5 and a maximum age of 15.3 years. The ClCr measured (median) by the Schwartz equation was 267 (IQR 199 - 320) mL/minute. The peak serum vancomycin level (estimated) was 21 (IQR 19 - 27) mg/L in the first hour, and the estimated trough level was 9 (IQR 6 - 11) mg/L in the sixth hour. Patients received the empirical dose normalized by the lowest weight, since the ideal weight was used for obese or overweight patients, while the actual weight was used for patients whose weight was below the ideal weight.

Regarding the division of patients into groups, as explained above, G1 consisted of 6 girls and 5 boys, with a median age of 3.0 years (IQR 1.7 - 4.5), a weight of 14 kg (IQR 10 - 16) and a median ClCr of 244 (IQR 228 - 274) mL/minute. G2 consisted of 3 girls and 8 boys, with a median age of 11.8 years (IQR 10.5 - 14.2), a weight of 42 kg (IQR 32 - 45) and a median ClCr of 318 (IQR 190 - 372) mL/minute (Table 1).

**Table 1:** Demographic characteristics by group

	G1	G2
Male sex/total	5/11	8/11
Age (years)	3.0 (1.7 - 4.5)	11.8 (10.5 - 14.2)
Weight (kg)	14 (10 - 16)	42 (32 - 45)
ClCr (mL/min)	244 (228 - 274)	318 (190 - 372)

G - group; ClCr - clearance of creatinine. The results are expressed as the total n/n or median (interquartile range).

**Figure 2:**
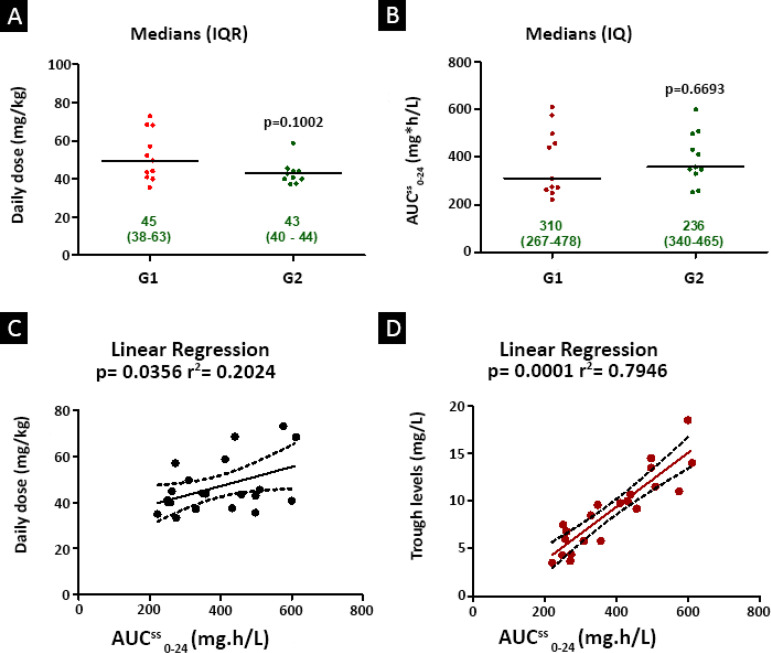
Comparison between groups and linear correlation of pharmacokinetic/pharmacodynamic parameters. Mann-Whitney, Prism 5.0: data expressed as medians (interquartile range), significance level p < 0.05. (A) Daily dose mg/kg. (B) Area under the curve (0 - 24 hours). Pearson correlation test, r^2^: linear correlation coefficient. Data are expressed as the mean (95% confidence interval), statistical significance p < 0.05. (C) Area under the curve (0 - 24 hours) *versus* daily dose; r^2^ = 0.2024, p = 0.0356. (D) Area under the curve (0 - 24 hours) *versus* trough concentration; r^2^ = 0.7953, p < 0.0001. AUC^ss^ 0_-24_ - area under the serum vancomycin concentration curve over the period of 0 - 24 hours. IQR - interquartile range; AUC - area under the curve.

The dose regimen for vancomycin in the study patients was determined based on the lowest weight of each patient; it was 45 (38 - 63) mg/kg/day for G1 and 43 (40 - 44) mg/kg/day for G2, with no significant difference (p = 0.1002) between the groups (Figure 2A). Regarding the AUC^ss^_0-24_, there was also no significant difference (p = 0.6693) between the groups: the value for G1 was 310 (267 - 478) mg x h/L, and for G2, it was 236 (340 - 465) mg x h/L. The linear correlation between the AUC and the serum vancomycin level was good, but the linear correlation obtained when the AUC was plotted against the daily dose normalized by body weight was low (Figure 2).

The PK/PD approach expressed by the AUC^ss^_0-24_/MIC showed that only 46% of the 22 patients were covered against pathogens with an MIC of 1mg/L, and no patient was covered against gram-positive pathogens with an MIC ≥ 2mg/L.

**Figure 3:**
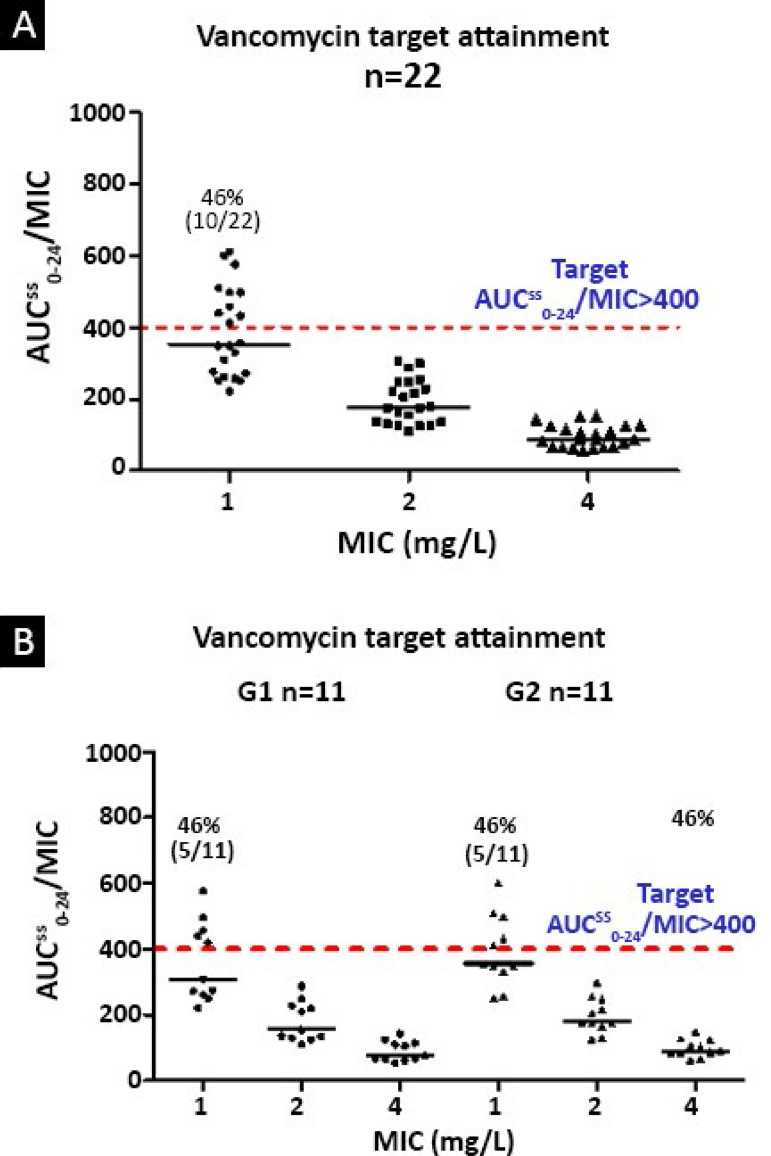
Percentage of patient coverage according to the minimum inhibitory concentration of the gram-positive bacteria. (A) Probability of target attainment for septic patients after the administration of an empirical dose of vancomycin. Dose: 44 (40 - 52) mg/kg/day; age: 9.5 (3.5 - 11.8) years, n = 22; median (interquartile range). (B) Pediatric patients divided into two groups based on age: G1: 3.0 (1.7 - 4.5) years, n1 = 11; G2: 10.8 (0.5 - 14.2) years, n2 = 11. PTA - probability of target attainment; AUCss0-24: area under the serum vancomycin concentration curve during the period of 0 - 24 hours; MIC - minimum inhibitory concentration.

When G1 and G2 are compared, it appears that the empirical doses of vancomycin were similar for both groups of patients. It is important to note that after the empirical dose was administered, coverage for all patients in both groups against pathogens with an MIC of 0.5mg/L was ensured; coverage against pathogens with an MIC of 1mg/L was increased to 5/11 of patients in both groups (Figure 3).

**Figure 4:**
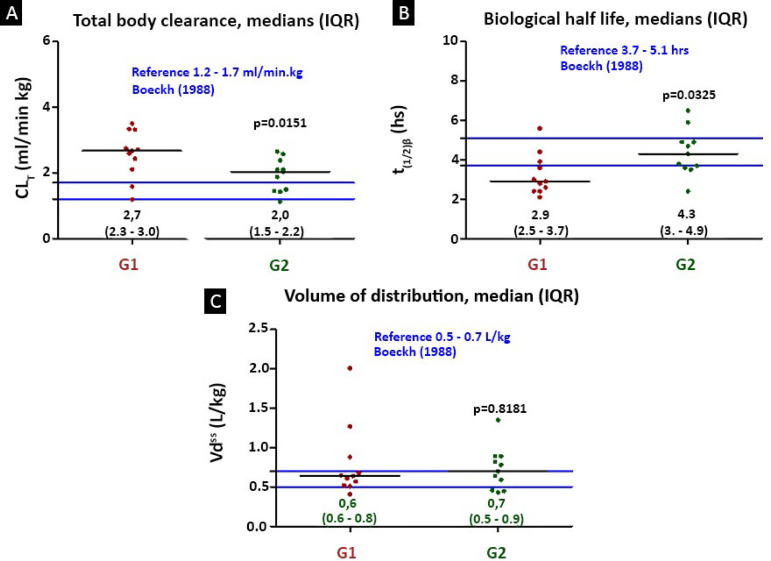
Changes in the pharmacokinetics of vancomycin in septic pediatric patients compared to the reference values reported in healthy volunteers.^(11)^ (A) Mann- Whitney, Prism 5.0: median (interquartile range), significance p < 0.05. (B) Pearson’s correlation test r^2^ linear correlation coefficient: means (95% confidence interval); significance level was set at p < 0.05. CL_T_ - total plasma clearance; Vd^ss^ - apparent volume of distribution; IQR - interquartile range.

The pharmacokinetics of vancomycin were altered due to the increase in CL_T_ and the shortening of the biological half-life in both study groups. The CL_T_ was significantly higher (p = 0.015) in G1 than in G2; it was 2.7mL/kg.min (IQR 2.3 - 3.0) in G1 and 2.0mL/kg.min (IQR 1.5 - 2.2) in G2. For the biological half-life, the pattern was inverse: 2.9 hours (IQR 2.5 - 3.7) in G1 and 4.3 hours (IQR 3.6 - 4.9) in G2, with p = 0.03. Regarding the volume of distribution, there was no difference between the groups. There was no linear correlation between the pharmacokinetic parameters despite the changes observed in clearance and biological half-life (Figure 4).

## DISCUSSION

According to the available consensuses, vancomycin is the first-choice antimicrobial agent for severe infections caused by gram-positive pathogens, especially *S. aureus* (MRSA).^(2,12)^ In these consensuses, data from adult patients are still being extrapolated to pediatric patients, with recommendations that the initial dose for pediatric patients be 40 - 60mg/kg/day to obtain a trough serum level of 15 - 20mg/L to obtain the desired clinical outcome. In the last 10 years, there has been a strong recommendation for therapeutic monitoring and titration of the vancomycin dose using the AUC^ss^_0-24_/MIC ratio instead of the trough serum concentration in septic patients in the ICU.^(2,3)^ According to this recommendation, the target to be achieved is AUC^ss^_0-24_/MIC > 400, and this index is considered the gold standard to ensure the effectiveness of vancomycin. Numerous studies in the literature suggest the serial collection of blood samples for the integration of the AUC of concentration over time. Most programs require the serial collection of three to five blood samples, such as the WinNonLin program (noncompartmental data analysis) applied in the PK/PD approach. In turn, the need to reduce the number of collection and the volume of blood collected is fundamental for individualized vancomycin therapy for pediatric patients in routine ICU care.^(13-15)^

Since the clearance of vancomycin is maximal in childhood and decreases with age, reduced serum levels of this antimicrobial are expected in pediatric patients compared to young adults and the elderly. However, recent studies indicate that the empirical daily dose of 40 to 45mg/kg has low coverage for any gram-positive agent, even those with a low MIC.^(9)^ It should thus be considered that the pediatric dose should start at 60mg/kg/day to reach the therapeutic target of AUC^ss^_0-24_/MIC > 400 against gram-positive bacteria with an of MIC 1mg/L.^(7-10,13-19)^

In our sample, the median initial dose of 44 mg/kg/day for vancomycin was estimated based on the lowest weight, and calculations between the actual and ideal weight were performed by the nutritionist of the ICU multidisciplinary team. However, this dose met the target (AUC^ss^_0-24_/MIC > 400) for gram-positive pathogens with an MIC of 1mg/L in only 46% of pediatric patients, a result that may be related to the higher clearance of the drug in patients with an increased ClCr (greater than 130mL/min/1.73 m^2^),^(20)^ as was the case for many patients in our study.

Based on the results obtained in the present study, it is suggested that the empirical daily dose of 60mg/kg be prescribed in patients with preserved renal function, in view of the evidence that lower doses do not cover patients against agents with an MIC of 1mg/L (the main target in intensive care, since an MIC of 2mg/L is considered resistant). The PK/PD approach based on the AUC^ss^_0-24_/MIC > 400 efficacy index is also recommended because dose adjustment based on the trough concentration has already been replaced by the PK/PD approach in more advanced centers, in addition to the fact that the use of vancomycin without plasma level monitoring should be discouraged.^(13,15,21)^

Thus, the assessment of vancomycin, in terms of both empirical therapy and the real-time adjusted dose, in early sepsis is cost-effective due to the ability to provide early medical intervention and to change the titration of the optimal dose for each pediatric patient being treated for severe infection caused by gram-positive agents in ICUs.^(9,13-15)^

Based on the reported data, our results showed that the patients in G1 and G2 received the same empirical dose normalized according to the lowest weight. Similar coverage was found based on the AUC^ss^_0-24_/MIC against pathogens with an MIC of 1mg/L; only 46% (5/11) of patients in both G1 and G2 were covered.

It is important to note that despite the similar coverage obtained for both groups, the CL_T_ was increased and the biological half-life was reduced in G1 compared to G2, justifying the need to increase the vancomycin dose in different proportions to meet the therapeutic target for treating isolated gram-positive pathogens in these patients. In other words, the younger the child is, the higher the prescribed daily dose must be to achieve the therapeutic target.

The limitations of our study include the small number of patients, the lack of follow-up to evaluate the microbiology and the clinical outcome. Additionally, according to Dhont et al.,^(22)^ the Schwartz formula may underestimate renal function. However, the calculations of renal clearance of the drug, its half-life and the AUC^ss^_0-24_ were based on the decrease in the serum level of the drug and the time required for it to occur and were not influenced by serum creatinine levels. The measurement of creatinine was important only to include patients with normal renal function in the study.

However, the objectives of this pilot study were achieved based on the standardization of collection, which allowed the use of the effectiveness prediction index (AUC^ss^_0-24_/MIC > 400) for vancomycin in pediatric patients admitted to the ICU with indications for antimicrobial use. Despite demonstrating that administering a daily dose closer to 40 mg/kg does not meet the therapeutic target against agents with an MIC of 1mg/L, inefficacy of lower doses was observed when the gold standard method was used. The next step is to correlate these findings with the agents isolated in cultures and with patient mortality.

## CONCLUSION

This single-center pilot study showed that less than half of children treated with vancomycin would be covered if infected by a gram-positive bacterium with an MIC of 1mg/L and that it is easy to calculate the ratio of the area under the serum vancomycin concentration curve to the minimum inhibitory concentration (gold standard). This allows individualization of the dose and the consequent need for finer adjustments. The younger the child, the greater the clearance of vancomycin is, and the shorter the half-life of the drug.

Our results suggest that the ideal initial dose of vancomycin is 60mg/kg/day for pediatric patients with preserved renal function, especially those younger than 7 years.
