# Supplementary material for: Does vancomycin administered at an empirical dose ensure coverage of pediatric patients against gram-positive pathogens?
Source: Rev Bras Ter Intensiva. 2020 Jul-Sep;32(3):391–7. doi: 10.5935/0103-507X.20200067 (PMC7595722; doi:10.5935/0103-507X.20200067)
Supplement: Supplementary file 1 [file rbti-32-03-0391-suppl01.pdf]

Does vancomycin administered at an empirical dose ensure coverage of pediatric patients against gram-positive pathogens?

A vancomicina administrada na dose empírica garante a cobertura de pacientes pediátricos contra patógenos Gram-positivos?

Frederico Ribeiro Pires[1], Stefano Ivani de Paula[1], Artur Figueiredo Delgado[1], Werther Brunow de Carvalho[1], Nilo José Coelho Duarte[2], Ronaldo Morales Júnior[3], Silvia Regina Cavani Jorge Santos[3]

Table 1S - Characterization of the study population

	Age (years)	Sex (F/M)	Ideal weight (kg)	Height (cm)	PIM 3	Probable source of infection	Use of VAD (dose)	Ventilatory support	Cr collection	ClCr	Trough 6 hours (mg/L)	AUC 24 (mg.h/L)
**Group 1**												
MVPS	4.4	F	17.5	109	0.2	Abdominal	None	HFC	0.22	273	10.7	440
PDB	4.1	F	14.5	91	0.4	Pulmonary	None	HFC	0.21	238	5.8	310
YFS	3.0	F	13.8	95	9.4	Skin	None	None	0.20	261	9.2	458
LGSS	1.7	F	7.8	71	0.2	Pulmonary	None	MV	0.16	244	4.3	250
SR	3.3	M	13.7	94	6.2	Abdominal	None	None	0.16	323	11.0	576
RROC	6.4	M	24.0	124	7.4	No apparent source	Adrenaline(0.3)	Oxygen	0.29	235	4.4	275
JLN	2.8	F	9.8	80	3.6	Central nervous system	None	Oxygen	0.20	220	6.8	262
NAV	6.6	F	20.5	115	32.9	Skin	Noradrenaline (0.6)/ milrinone (0.75)	MV	0.41	154	14.0	612
HRF	1.5	M	10.3	80	11.4	Blood stream	None	MV	0.16	275	3.5	222
EBO	1.7	M	9.0	75	3.6	Blood stream	None	MV	0.26	159	14.5	498
LMSP	4.5	M	14.0	96	9.4	Urine	None	HFC	0.19	278	3.7	272
**Group 2**												
KRN	13.8	M	45.5	155	12.3	Pulmonary	None	NIV	0.27	402.	9.6	349
KBO	14.0	M	41.4	150	0.6	Central nervous system	None	Oxygen	0.27	402	9.6	349
KRM	11.2	F	42.5	156	0.5	No apparent source	None	None	0.27	318	10.0	432
LEGPR	9.8	F	32.3	140	2.8	No apparent source	None	HFC	0.40	193	8.5	330
WVJ	14.6	M	50.5	161	4.3	Skin	None	None	0.66	171	18.5	600
SMJ	10.0	M	32.0	140	9.2	Abdominal	None	None	0.24	321	5.8	358
BNAC	11.0	M	24.5	119	18	Skin	None	MV	0.18	364	6.0	258
VAS	14.7	M	50.0	161	8.8	Abdominal	None	None	0.38	297	7.5	252
GOCB	11.8	M	34.0	140	11.3	Blood stream	None	MV	0.17	453	9.8	412
APSS	15.3	F	52.0	160	7.6	No apparent focus	None	None	0.64	175	13.5	498
MGS	9.3	M	30.7	143	7.7	Pulmonary	Milrinone(0.5)	MV	0.42	187	11.5	510

F - female; M - male; PIM 3 - Pediatric Index of Mortality 3; VAD - vasoactive drug; Cr - creatinine; ClCr - creatinine clearance; AUC - area under the curve; HFC – high-flow catheter; MV - invasive mechanical ventilation; NIV – noninvasive ventilation. Dose of vasoactive drug in mcg/kg/minute.
